# Stabilization of mid-sized silicon nanoparticles by functionalization with acrylic acid

**DOI:** 10.1186/1556-276X-7-76

**Published:** 2012-01-16

**Authors:** Robert Bywalez, Hatice Karacuban, Hermann Nienhaus, Christof Schulz, Hartmut Wiggers

**Affiliations:** 1 IVG, Institute for Combustion and Gasdynamics, University Duisburg-Essen, Duisburg, 47048, Germany; 2 Faculty of Physics, University Duisburg-Essen, Duisburg, 47048, Germany; 3 CeNIDE, Center for Nanointegration Duisburg-Essen, Duisburg, 47048, Germany

## Abstract

We present an enhanced method to form stable dispersions of medium-sized silicon nanoparticles for solar cell applications by thermally induced grafting of acrylic acid to the nanoparticle surface. In order to confirm their covalent attachment on the silicon nanoparticles and to assess the quality of the functionalization, X-ray photoelectron spectroscopy and diffuse reflectance infrared Fourier spectroscopy measurements were carried out. The stability of the dispersion was elucidated by dynamic light scattering and Zeta-potential measurements, showing no sign of degradation for months.

## Introduction

Silicon nanoparticles received considerable attention in recent years, especially since the discovery of quantum-confined luminescence in silicon. Besides optoelectronic devices [1,2], silicon nanoparticles are envisioned for a much broader range of applications, especially if they can be processed by printing techniques. Future generations of lithium-ion batteries might rely on printable silicon nanoparticles as a part of the electrode setup, boosting the battery's capacity [3,4]. Furthermore, their potential in photovoltaics is shifting into the focus of interest. For example, silicon nanoparticles were used as a top layer on commercial polycrystalline solar cells boosting their power performance by 60% in the blue/UV range and also as a principal component of a heterojunction solar cell in combination with P3HT [5,6]. The reported top efficiencies of 1.15% are promising although the specimens need to be stored under inert conditions.

One of the basic requirements for the industrial applicability of silicon nanoparticles is the availability of printable dispersions, and in cases of electronics applications, a suitable protection against oxidation. The most common approach is to functionalize the particles with various organic substances like alkenes [7-9], amines [10], and phospholipids [11]. Although it has been shown that this leads to fairly stable dispersions of small nanoparticles with sizes below 5 nm, the situation gets more complicated when dealing with particles of larger sizes. Veinot et al. showed a strong size dependence of hydrosilylation efficiency for silicon nanoparticles. Particles with 5 to 7 nm in diameter required significant longer reaction times than the particles with 2 to 3 nm in diameter and still showed worse functionalization efficiencies [12]. These effects are attributed to changes in reaction chemistry. Together with the observation that smaller nanoparticles require a lower degree of surface grafted molecules [13] to form stable dispersions and the fact that the decreasing surface curvature of large nanoparticles reduces the specific surface coverage [14], it is obvious that functionalization of mid-sized silicon nanoparticles is challenging. While the functionalization with alkenes, as also established in our group, yields stable dispersions from small nanoparticles with sizes below 5 nm [7], the same reaction routes do not lead to stable dispersions with larger particles.

A surface coverage with acrylic acid molecules was used to render our particles hydrophilic and provide stable dispersions even for particles exceeding a few nanometers in diameter. Li et al. and He et al. [15,16] used UV-grafted polyacrylic acid to render small nanoparticles water soluble. Sato et al. [17] also used a similar approach on silicon nanoparticles of < 2 nm in diameter to provide a termination using acrylic acid. Nevertheless, there is no sound evidence for the covalent attachment of acrylic acid via a Si-C bond, and polymerization cannot be excluded. The rather high oxidation levels, although only small particles were used, indicate low (insufficient) functionalization efficiencies. Not only surface oxidation, but also ligands with a long chain length as well hamper the applicability of silicon nanoparticles in electric or electroluminescent devices because they prevent an efficient charge transport compared to short ones [18]. Therefore, short functionalization chain lengths are desired as they ensure better charge transport compared to their larger counterparts [18].

The approach used in this work reduces the thickness of the surface coating compared to commonly used *n*-alkenes and polymers. We present a fast functionalization route for medium-sized particles of a few 10 nm in diameter, with sound dispersion properties as well as very low oxygen content. Furthermore, clear evidence for the underlying binding mechanism is provided.

## Experimental details

### Materials

Hydrofluoric acid (40%), methanol, acrylic acid, and isopropanol were purchased from VWR International, Darmstadt, Germany and used as received.

### Synthesis and functionalization

**Figure 1:**
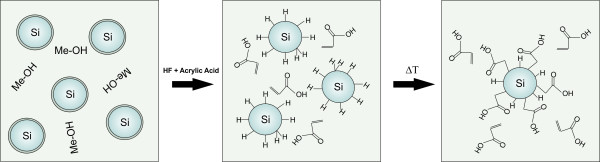
**Expected reaction scheme**. As-prepared silicon nanoparticles are dispersed in methanol (left) and etched with hydrofluoric acid for 20 min. After the filtration process, the hydrogen-terminated particles are transferred into the acrylic acid (middle) and heated up to 80°C for 25 min to finish the functionalization (left).

Spherical, single crystalline silicon nanoparticles were synthesized in a microwave plasma reactor; details concerning the method can be found in the study of Petermann et al. [19]. This method permits a cost-effective, large-scale production of silicon nanoparticles with production rates of up to a few 10 g/h. The particles are covered with a native oxide shell of 1 to 2 nm when stored in ambient conditions. The average diameter of the particles used for this study is 37 nm (calculated from their specific surface area assuming spherical, monodisperse particles), whereas the count median diameter extracted from transmission electron microscopy [TEM] measurements has a value of 41 nm with a geometrical standard deviation of 1.37 [see Additional files 1 and 2]. This particle size is quite large compared to most studies conducted on functionalized silicon nanoparticles [7,10,11]. In order to prepare the particles for electronic applications, the oxide shell has to be removed and the particles need to be functionalized to prevent them from reoxidation. Three hundred thirty milligrams of silicon nanoparticles were dispersed in methanol and etched with 20 ml of hydrofluoric acid for 25 min in a nitrogen-filled glove box. The etching solution was filtrated, and the particles were conveyed into 20 ml of acrylic acid, heated to 80°C, and left to react for 25 min. Due to the formation of a silicon-carbon bond, the alkene group of the acrylic acid changes from a double to a single bond, leading to propionic acid-coated silicon nanoparticles. The reaction scheme is depicted in Figure 1. Afterwards, the particles were filtered again, washed with chloroform, and centrifuged out of chloroform dispersion. Finally, the particles were dried overnight. To alleviate particle handling in the X-ray photoelectron spectroscopy [XPS], approximately 15 mg of the nanoparticle powder was pressed into a pellet. Dispersions were formed by introducing particles in isopropyl alcohol and subsequently sonicated for 20 min. Isopropyl alcohol was chosen because of its advantages for printing that are mainly due to its high vapor pressure in comparison to water. A polyacrylic reference sample was formed by heating acrylic acid at 120°C for 80 min.

### Characterization

Particle diameters were calculated from Brunauer, Emmett, and Teller [BET] specific surface measurement with Quantachrome Nova 2200 (Quantachrome Instruments, Boynton Beach, FL, USA) and TEM with a FEI Tecnai F20 ST microscope (FEI Co., Hillsboro, OR, USA). Surface functionalization was confirmed via diffuse reflectance infrared Fourier transform spectroscopy [DRIFTS] utilizing a Bruker IFS66v/S spectrometer (Bruker Optik GmbH, Ettlingen, Germany), and XPS was done with a SPECS Phoibos 100 spectrometer (SPECS GmbH, Berlin, Germany). Dispersion quality and stability were probed via dynamic light scattering [DLS] and Zeta-potential measurements, both performed with a Malvern Nano ZS instrument (Malvern Instruments, Worcestershire, United Kingdom).

## Results and discussion

**Figure 2:**
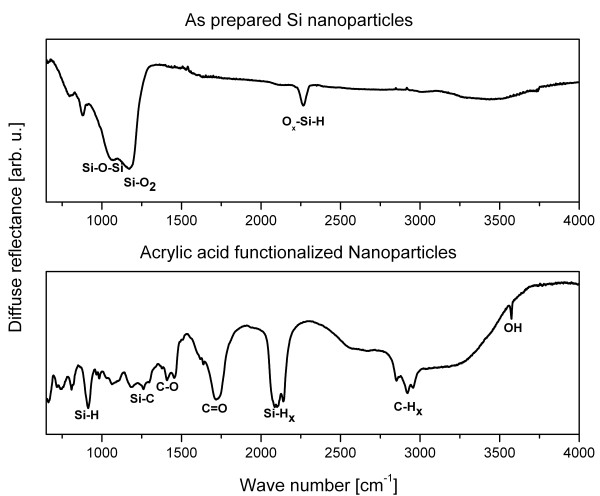
**DRIFT spectra**. DRIFT spectra of as-prepared nanoparticles (top), and acrylic acid-functionalized nanoparticles (bottom) with the appointed functional groups.

A comparison of the DRIFTS spectra of the as-prepared particles and the functionalized ones (*cf*. Figure 2) shows the strong SiO_x _absorption signal at 1,000 to 1,180 cm^-1 ^for the as-prepared particles. It includes the Si-O-Si vibration at 1,050 cm^-1 ^and the SiO_2 _absorption around 1,158 cm^-1 ^[20] and disappears for the functionalized sample, indicating that almost all silicon oxide was removed. The strong signal at 1,720 cm^-1 ^originates from the C = O out-of-phase vibration, which together with the very broad OH band centered around 3,150 cm^-1 ^identifies the attached molecules as acrylic acid [21]. The C-CH_x _vibrations at 2,956, 2,922, and 2,852 cm^-1 ^further strengthen this result. An interesting hint is provided by the sharp peak at 3,574 cm^-1 ^that indicates that carboxylic acid monomers are present [21,22]. This OH-stretch vibration does not appear in the liquid phase because dimer and oligomer configurations cause this vibration to vanish [22]. The prominent Si-H_x _vibrations located around 2,097 cm^-1^, along with the SiH_2 _scissor mode or the SiH_3 _degenerate deformation vibration at 902 cm^-1^, point out that it is not possible to completely cover the particles with acrylic acid.

In order to help distinguish the surface termination from polyacrylic acid, the respective Fourier transform infrared [FTIR] spectra are shown as well [see Additional file 3]. The most striking difference here is the missing OH-vibration around 3,574 cm^-1^.

The Si-CH_2 _scissoring vibration at 1,450 cm^-1 ^is regularly used as an indicator for the covalent attachment of the functionalization agent onto the molecules [10,17,23]. This choice is problematic because the strong C-CH_x _vibration signal appears in the same frequency range and overlaps with the Si-C signal [21,24]. A more reasonable selection is the Si-CH_2 _stretching vibration at 1,259 cm^-1^, *cf*. Figure 2. However, this vibration is weak and hardly detectable, as could be seen in the work of Rosso-Vasic et al. [23].

**Figure 3:**
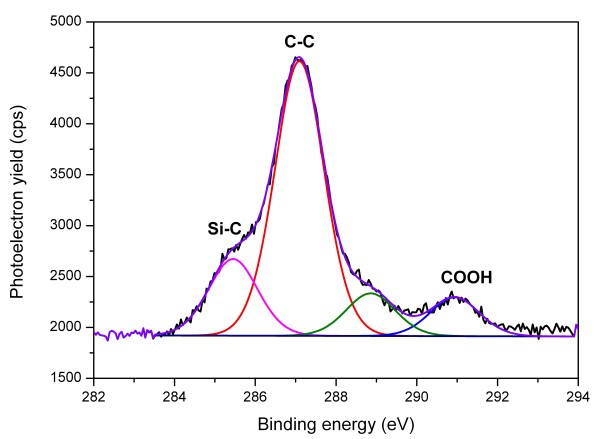
**XPS C 1*s *spectra of acrylic acid-functionalized nanoparticles**. The maximum of the C-C peak is lying at 287.1 eV, and the Si-C peak is centered at 285.4 eV, displaying a shift of 1.7 eV. The peak attributed to the carboxyl group is located at 291 eV.

**Figure 4:**
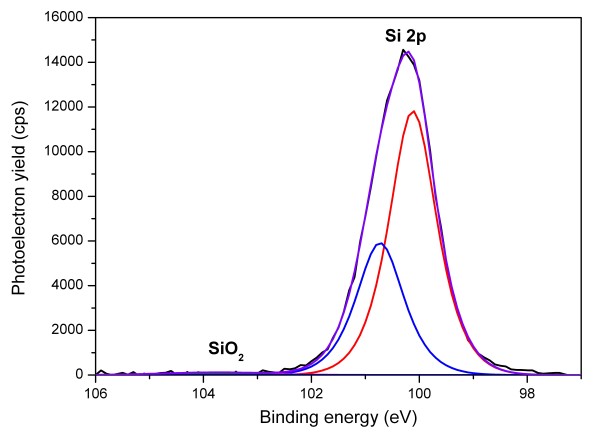
**XPS Si 2*p *spectra of the functionalized nanoparticles**. The central silicon peak is a superposition of the Si 2*p*_1/2 _and the Si 2*p*_3/2 _signals. The energy range in which the silicon oxide peak is expected to show is implied.

XPS measurements were performed to unambiguously prove the formation of a covalent bond between the acrylic acid and the silicon nanoparticles. Observing the C 1*s *signal of the XPS spectra as displayed in Figure 3, the most prominent peak at 287.1 eV originates from the C-C bonds of the acrylic acid and is slightly shifted as reported in literature [25], while the shoulder centered around 285.4 eV can be attributed to the Si-C bond by displaying the characteristic shift from the main carbon C-C peak [25]. This clearly indicates that the acrylic acid molecules are chemically bonded to the surface via a covalent Si-C bond, resulting from the reaction of the hydrogen-terminated silicon surface and the alkene group of the acrylic acid. This, together with the missing Si-O vibration from FTIR, rules out that covalent bonding via oxygen took place as it is known to occur with UV-initiated reactions [16]. The signal of the carboxylic group is also present at 290.0 eV, displaying a shift from the main C 1*s *peak in agreement with the findings of Li et al. [15], thus solidifying the successful attachment of acrylic acid. A comparison between the peak intensities of the Si-C and C-C signals further stresses the assumption that surface grafting with mostly acrylic acid monomers took place; however, it is not possible to completely exclude any oligomerization of a few monomers. As can be seen in the DRIFT spectra (*cf*. Figure 2), the functionalized particles show hardly any sign of oxidation. This is quite surprising due to the fact that the alkyl functionalization on particles of similar size often showed immediate reoxidation [26] which was attributed to an incomplete surface coverage. Regardless that the surface coverage of our material is also incomplete, oxidation seems inhibited. To underpin those results, an XPS analysis of the Si 2*p *signal was performed. As can be seen in Figure 4, the particles are practically oxygen-free as no silicon oxide signal is observable. While the SiO_2 _signal is expected to be shifted 3.45 eV towards higher binding energies from the main Si 2*p *peak, the substoichiometric SiO_x _signal is located between 1 and 2 eV below that for silicon dioxide [27]. Due to the fact that all sample preparation and post processing, except the functionalization itself, took place in ambient conditions, this is a remarkable finding. We attribute this partially to the fact that the functional carbonyl groups sticking out of the particle surface may form interconnecting hydrogen bonds and also hydrogen bonds with other polar molecules such as water or additional acrylic acid, preventing the particle surface from immediate reoxidation.

**Figure 5:**
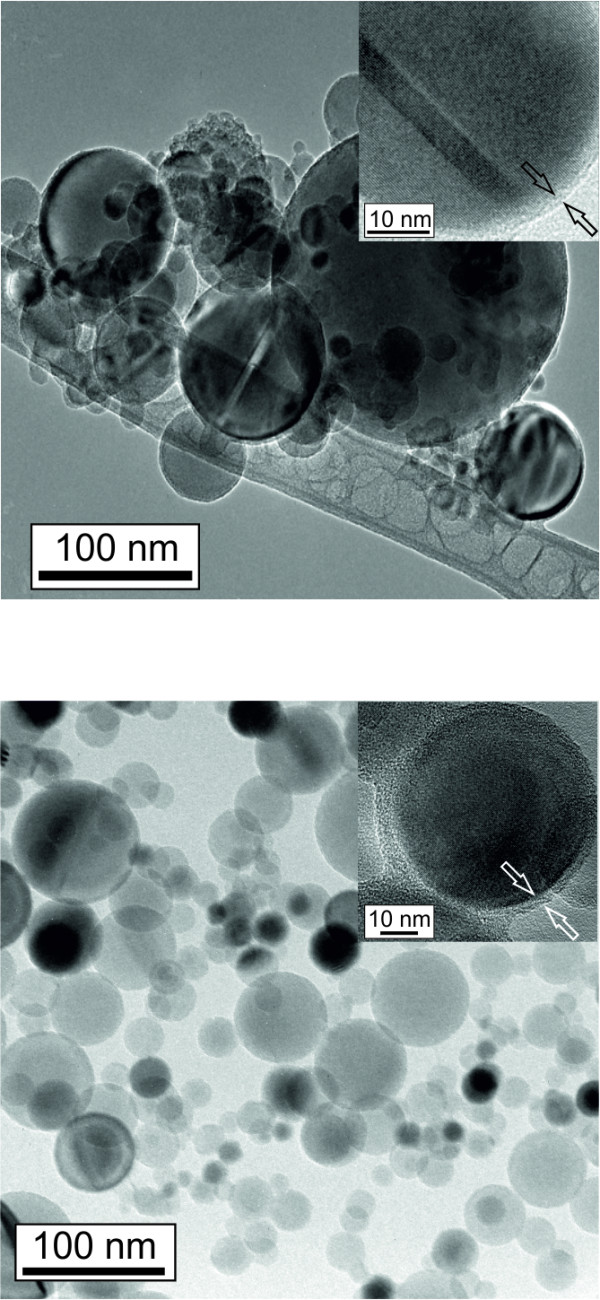
**TEM pictures of as-prepared (bottom) and functionalized Si nanoparticles (top) with high-resolution insets**.

To visualize the nature of the functionalization and to compare the as-prepared and functionalized silicon nanoparticles, TEM measurements were performed. Figure 5 shows the particles before and after surface treatment. The as-prepared particles are covered with a native oxide shell, as can be seen in the high-resolution inset. The functionalized particles were extracted from a dispersion made from isopropanol. A formation of soft agglomerates is observed; however, the TEM image clearly shows that each particle is covered with an individual shell of approximately 1 nm in diameter. The particles are coated with a thin dense layer which remains unchanged irrespective whether the particles are freshly functionalized or stored for 10 months in isopropanol. In case of polymerization during functionalization, one would expect a polymer host structure containing several, statistically distributed nanoparticles [16]. In the inset, a higher magnification of an individual particle coating is provided. As known from FTIR and XPS results, a surface coverage with silicon oxide can be excluded. Therefore, we suppose that the surface is covered with both monolayer as well as bilayer of acrylic acid and short oligomers. That would be in agreement with all the presented results and can explain the dense-looking shell capping of our particles. This unusual surface termination can be responsible for the very low oxidation level and provides a quite good protection against oxidation, especially in dispersions.

**Figure 6:**
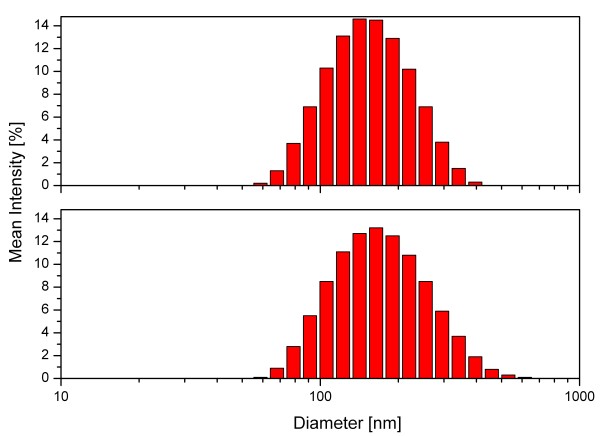
**DLS measurement of as-prepared acrylic acid-functionalized nanoparticles (top) and after 7 weeks (bottom)**.

The properties of the dispersions of the functionalized silicon nanoparticles were investigated by DLS and Zeta-potential measurements. Measurements were taken from the freshly dispersed particles and compared to the same dispersion after 7 weeks (see Figure 6). The size distribution of the 7-week-old dispersion is nearly identical to that of the as-prepared one. A Zeta potential of -72.9 mV was measured which indicates a highly stable dispersion. The combination of DLS and Zeta-potential data provides evidence of a long-term dispersion stability.

## Conclusion

Highly stable dispersions of silicon nanoparticles stabilized by acrylic acid were formed. Evidence for dispersion stability was provided by DLS and Zeta-potential measurements. FTIR and XPS measurements were used to assess the functionalization quality and elucidated the binding mechanism between acrylic acid and silicon nanoparticles. TEM images provided further insights into the nature of the surface termination. Future experiments will focus on the electrical properties of functionalized particles as well as printed layers.

## Competing interests

The authors declare that they have no competing interests.

## Authors' contributions

RB functionalized the particles, carried out the FTIR and DLS measurements, drafted the manuscript, and participated in the design of experiments. HK and HN carried out the XPS measurements and interpretation and contributed to the discussion of the results. HW and CS were involved in the scientific guidance of the research, the discussion of the experimental results, and in revising the manuscript. All authors read and approved the final manuscript.

## Supplementary Material

Additional file 1 **Supporting information**. Data on the multi-point BET summary. Click here for file

Additional file 2 **Support particle size distribution**. A graph showing support particle size distribution. Lognormal size distribution of the Silicon nanoparticle ensemble as calculated from the TEM pictures. The Geometric standard deviation is 1,37. Click here for file

Additional file 3 **FTIR supplemental image**. FTIR spectra of as prepared silicon Nanoparticles (top), acrylic acid functionalized Si NPs (middle), and a polyacrylic acid reference sample (bottom), with the assigned group frequencies. Click here for file
