# Supplementary material for: Stabilization of mid-sized silicon nanoparticles by functionalization with acrylic acid
Source: Nanoscale Res Lett. 2012 Jan 16;7(1):76. doi: 10.1186/1556-276X-7-76 (PMC3312836; doi:10.1186/1556-276X-7-76)
Supplement: Additional file 1 — Supporting information. Data on the multi-point BET summary. [file 1556-276X-7-76-S1.DOCX]

Supporting information

**Multi Point BET** Summary:

Slope: 49,785

Intercept: 9,446 e^-1^

Correlation coefficient, r: 0,999920

C constant: 53,703

Surface Area: 68.649 m^2^/g
