# Supplementary figures and images for: Stabilization of mid-sized silicon nanoparticles by functionalization with acrylic acid
Source: Nanoscale Res Lett. 2012 Jan 16;7(1):76. doi: 10.1186/1556-276X-7-76 (PMC3312836; doi:10.1186/1556-276X-7-76)

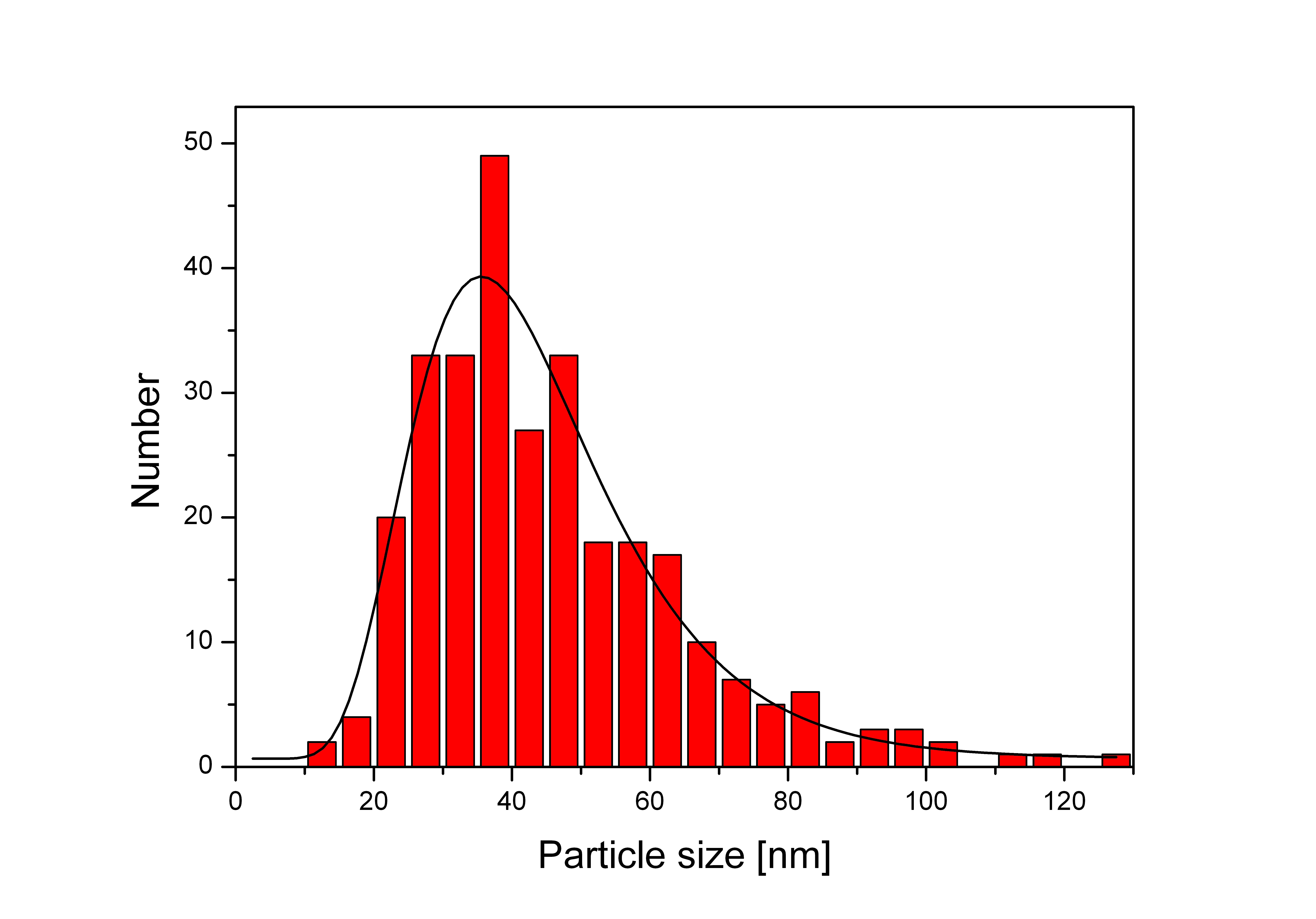

Supplement: Additional file 2 — Support particle size distribution. A graph showing support particle size distribution. Lognormal size distribution of the Silicon nanoparticle ensemble as calculated from the TEM pictures. The Geometric standard deviation is 1,37. [file 1556-276X-7-76-S2.JPEG]

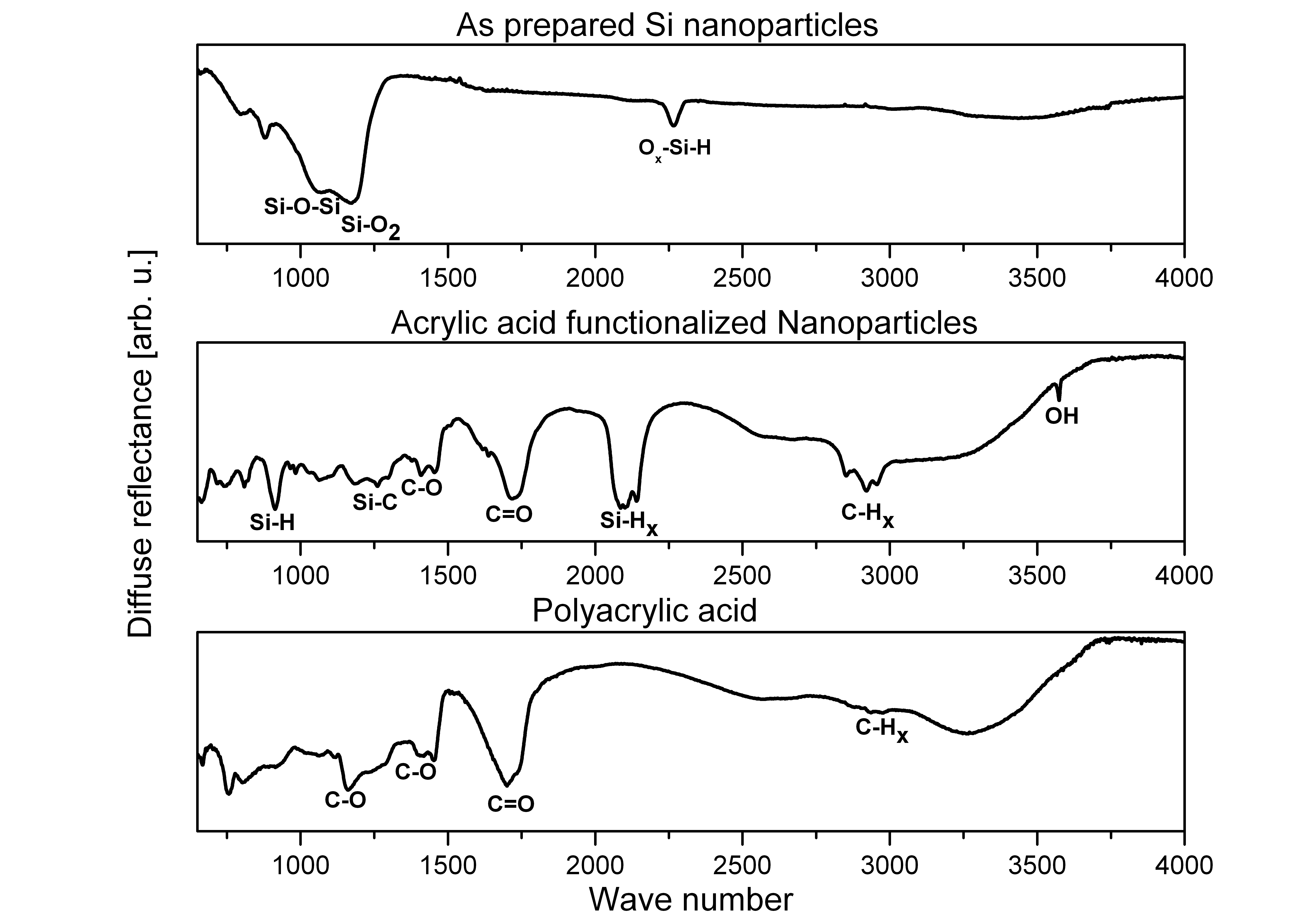

Supplement: Additional file 3 — FTIR supplemental image. FTIR spectra of as prepared silicon Nanoparticles (top), acrylic acid functionalized Si NPs (middle), and a polyacrylic acid reference sample (bottom), with the assigned group frequencies. [file 1556-276X-7-76-S3.JPEG]
